# Saxophone penis due to primary lymphoedema

**DOI:** 10.4103/0971-9261.59611

**Published:** 2009

**Authors:** Vishal K. Jain, Sangram Singh, Saurabh Garge, Anupama Negi

**Affiliations:** Department of Surgery, SAIMS, Indore, India; 1 Department of Paediatric Surgery, SAIMS, Indore, India

**Keywords:** Deformity, lymphoedema, penis, saxophone penis

## Abstract

Congenital lymphoedema is a rare disorder that may result in disfiguring edema of the male genitalia. The treatment of persistent lymphoedema is surgical and consists of meticulous excision of all subcutaneous layers of the affected skin, combined with reconstruction of the penis and or scrotum.

## INTRODUCTION

Primary congenital lymphedema is the rarest form of primary lymphedema. Lymphedema of the extremities presents at birth and rarely involves the genitalia. Primary penile lymphedema occurs infrequently and is seen in conjunction with a similar process in the scrotum. The accepted form of treatment is surgery since conservative medical treatment is of little value. Different surgical techniques have been described, but no single procedure has emerged as the ideal treatment.

## CASE REPORT

**Figure 1:**
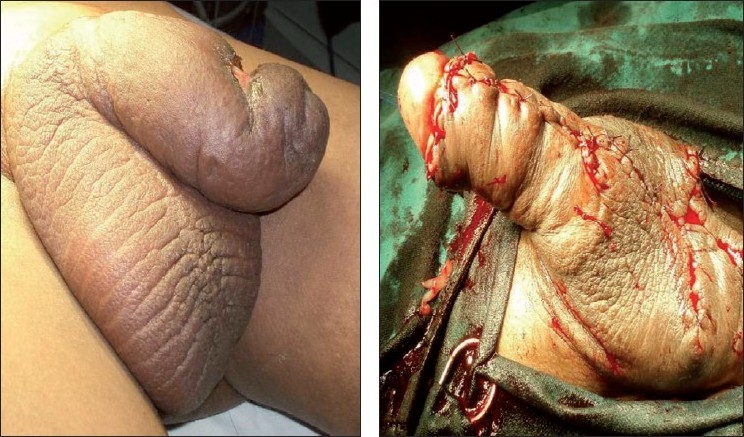
Pre- and post-operative photograph of saxophone penis

We report a case of scrotal and penile lymphoedema in a 12-year-old child since 5 years. He did not have any history of trauma, infection, or any other cause of secondary lymphoedema. Clinically, he did not have lymphadenopathy. General and systemic examinations were essentially normal. The lower limbs were normal with no signs of lymphoedema. Genital examination revealed a cold, non-tender, large-curved penis measuring 5 inches in length and 5 inches in circumference, looking like a “saxophone” [Figure 1]. There was evidence of an ulcer. It was a 4 × 3 cm ulcer with slough and pale granulation tissue at its floor and indurated margins. The scrotum was huge and its contents could barely be palpated. Transillumination test of scrotal swelling was negative.

Routine urinalysis, hematological, and blood biochemical tests were normal. Ultrasonography of testes and lower urinary tract were normal. Serologic study was negative for filariasis. With the diagnosis of primary lymphoedema of penis and scrotum, surgery was planned in which extensive resection of the involved tissue, scrotoplasty by the use of skin flaps of 1/3 of the posterior scrotum, and total excision of the lymphedematous tissue of the penile shaft with cosmetic reconstruction without skin grafting. In this case, a circumferential incision was made 5–10 mm from the coronal sulcus and deepened to the level of Buck's fascia. The skin and subcutaneous tissue were then completely dissected away from the penis. The skin was everted and all of the abnormal lymphedematous tissue excised up to the dermal skin margin. The skin was then tailored to the size of the penile shaft and re approximated. A catheter was placed intra-operatively at the end of surgery. This method was employed with the advantages of (1) shorter hospitalization, (2) lack of morbidity associated with the skin donor site, and (3) satisfactory cosmetic results. Histopathologic examination showed nonspecific chronic inflammation with areas of epidermal thickening and dermal fibrosis. Wound healed primarily and sutures were removed on 14th postoperative day.

## DISCUSSION

Lymphedema is the occurrence of chronically swollen extremities or rarely the genitals due to inadequate drainage of interstitial fluid by the lymphatics. Lymphedema is of two types: primary and secondary. Primary lymphedema is uncommon and has female predominance. It may be congenital or familial (Milroy's disease) or idiopathic appearing either at puberty (precox), or after 35 years of age (tardum).[1,2]

Penile and scrotal lymphedema mostly occurs following an infection or as a reaction to trauma. Idiopathic lymphedema is rarely seen and is caused by a primary obstruction of lymphatic vessels of scrotum.[3]

Genital elephantiasis is a functionally disabling and emotionally incapacitating entity. Lymphedema of the male genitalia is caused by reduced lymphatic flow, with subsequent enlargement of the penis and scrotum. Swelling is characterized by severe discomfort, limitations of local hygiene and ambulation, and a progressive loss of sexual and urinary function. These functional disabilities cause extreme emotional stress making surgical intervention imperative.[9] Congenital lymphedema of the genitalia has profound physical and psychological consequences for the growing child. Extensive resection of this tissue and reconstruction by skin grafting offers a less than satisfactory cosmetic result.

No effective medical treatment has been introduced; however patient with spotty occurrence of genital lymphedema may respond to prolonged course of fluoroquinolones. Different surgical methods for the treatment of chronic genital lymphedema have been reported in the literature. Two main methods are as follows:

1. Physiologic methods or lymphangioplasty through which lymphatic discharge from involved regions to new lymphatic channels is obtained.
2. Lymphangiectomy with reconstructive surgery.[4]

Lymphangioplasty is used in the cases of recurrent lymphedema; however, this method can not be successful in the cases of chronic fibrosis or lymphedema caused by radiation because of the lack of appropriate lymphatic channels.[4]

Various methods of reconstruction of genital elephantiasis involve excision of affected tissue and its reconstruction with or without lymphangioplasty. Out of the several procedures described in the literature, modified Charles procedure looks most promising. These surgical procedures if performed well give remarkably good cosmetic results with tremendous improvement in quality of life of these unfortunate patients with genital elephantiasis.[9]

Lymphangiectomy includes the removal of superficial lymphatic network, which is located above the Buck's fascia which is derived from median raphae and prepuce lymphatics. These lymphatics drain to superficial posterior lymphatic channels. A deeper system is located beneath the Buck's fascia and is drained into deep inguinal lymph nodes.- The above method of drainage leads to the success of our surgical method.

It is essential to remove involved skin and subcutaneous tissue completely around the penis and the scrotum (reduction scrotoplasty) to prevent lymphedema recurrence followed by reconstructive surgery of penis and scrotum.[5,6] The posterior scrotal skin usually uninvolved and may be used as a source of skin flap for reconstruction of scrotum.

Surgical complications of elephantiasis or genital lymphedema include hemorrhage, hematoma, urethral injury, infection, painful erection, decrease of sensation, and scar in suture line. These complications could be reduced by using a proper incision, use of Z plasty instead of longitudinal suture, separating of testes, and cord by an external incision in scrotum before taking any measure, and removal of involved tissue.[4,8]
